# AI-derived facial redness score is associated with body mass index in a large Japanese screening cohort

**DOI:** 10.1016/j.jdin.2026.07.010

**Published:** 2026-08-06

**Authors:** Kimi Iinuma, Kazuyasu Fujii, Chisa Nakashima, Shunsuke Morita, Toshio Morikawa, Hidekazu Yamada, Ryuichi Morishita, Atsushi Otsuka

**Affiliations:** a Department of Dermatology, Kindai University Faculty of Medicine, Osaka, Japan; b Anti-Aging Center, Kindai University, Osaka, Japan; c Pharmaceutical Research and Technology Institute, Kindai University, Osaka, Japan; d Department of Clinical Gene Therapy, School of Medicine, Osaka University, Suita, Osaka, Japan

**Keywords:** AI-derived facial redness score, body mass index, digital phenotyping, epidemiology, obesity

*To the Editor:* Obesity is associated with systemic inflammation, but its relationship with quantitative facial skin phenotypes remains unclear.^1^^,^^2^ We examined whether body mass index (BMI) was associated with AI-derived facial redness in a Japanese screening cohort, treating the redness output as an image-derived score rather than dermatologist-assessed erythema.

This cross-sectional study used anonymized data from adults in the Karada Measurement program at the Osaka Healthcare Pavilion, Expo 2025 Osaka, Kansai, Japan. Of 484,462 individuals assessed, 74,078 were excluded for missing BMI (*n* = 50,293) or redness scores (*n* = 23,785), leaving 410,384 for analysis (Supplementary Fig 1, available via Mendeley at https://data.mendeley.com/datasets/np3r5kwfrx/1). BMI was categorized using Japanese criteria.^3^ An AI-based skin analysis system (Perfect Corp.) quantified 14 facial parameters on 1 to 100 scales; the redness score was reverse-coded (reverse-coded score = 101 - original score) so that higher scores indicated greater image-derived facial redness. Pearson correlations examined BMI and each continuous facial score. Logistic regression estimated age-adjusted odds ratios (ORs) for a high redness score (reverse-coded score ≥31), with age as a continuous covariate and normal weight as the reference. This cutoff was sensitivity-tested against 4 alternative thresholds, which yielded consistent associations (Supplementary Table I, available via Mendeley at https://data.mendeley.com/datasets/np3r5kwfrx/1). After sex- and age-related effect modification was observed, women aged 30-59 years and age-matched men were examined as exploratory subgroups.

The cohort comprised 410,384 adults (250,318 women and 160,066 men; mean age, 44.4 years; mean BMI, 22.3 kg/m^2^). Among 14 AI-derived parameters, redness showed one of the most positive correlations with BMI (overall r = 0.312; women, r = 0.236; men, r = 0.257; women aged 30-59 years, r = 0.284). Texture showed a correlation of comparable magnitude (r = −0.313; lower scores indicate more irregularities), although its stratified associations were less consistent.

Obesity class I and class II or higher were associated with higher odds of a high redness score than normal weight (age-adjusted OR, 3.15 [95% CI, 3.03-3.28] and 5.92 [5.61-6.25], respectively; Table Ⅰ). Associations were stronger in women aged 30-59 years (OR, 4.52 [4.14-4.94] and 10.57 [9.54-11.71]) than in age-matched men (OR, 2.20 [2.07-2.33] and 3.77 [3.48-4.10], respectively). The reverse-coded redness score was minimally correlated with vascular age (r = −0.014) and pulse rate (r = −0.035), arguing against these measures fully explaining the association.

**Table I:** Association between BMI and high AI-derived facial redness (reverse-coded redness score ≥31)

Group	Comparison	Reference group	Comparison group	Age-adjusted OR (95% CI)
Overall	Obesity class I vs normal	5,643/265,723 (2.1%)	4,625/70,978 (6.5%)	3.15 (3.03-3.28)
Overall	Obesity class II+ vs normal	5,643/265,723 (2.1%)	1,971/17,224 (11.4%)	5.92 (5.61-6.25)
Women 30-59 y	Obesity class I vs normal	1,115/105,799 (1.1%)	948/21,068 (4.5%)	4.52 (4.14-4.94)
Women 30-59 y	Obesity class II+ vs normal	1,115/105,799 (1.1%)	608/5998 (10.1%)	10.57 (9.54-11.71)
Men 30-59 y	Obesity class I vs normal	2,215/60,561 (3.7%)	2,266/28,682 (7.9%)	2.20 (2.07-2.33)
Men 30-59 y	Obesity class II+ vs normal	2,215/60,561 (3.7%)	896/7109 (12.6%)	3.77 (3.48-4.10)

Normal weight was BMI 18.5 to 24.9 kg/m^2^. Obesity classes were defined according to Japanese criteria.

*CI*, Confidence interval; *OR*, odds ratio.

This exploratory, cross-sectional analysis cannot establish causality or clinical actionability. The AI redness score was not validated against dermatologist-assessed erythema in this cohort, and its performance across skin tones, cosmetic use, sex, age, and BMI categories was not established. Importantly, the observed association may largely reflect a higher prevalence of rosacea and other inflammatory facial dermatoses among individuals with obesity^4^ rather than a novel cutaneous phenotype; because the dataset was fully anonymized, these diagnoses could not be adjudicated. Alcohol consumption and the ALDH2 rs671 polymorphism, which causes pronounced alcohol-induced facial flushing in roughly 40% of East Asians,^5^ were also unmeasured and may confound the association. Makeup, phototype, photodamage, medications, hormonal factors, ultraviolet exposure, and image-acquisition effects were likewise unadjusted. AI-derived facial redness may thus be an image-based phenotype associated with BMI in this screening population, but prospective studies incorporating clinical skin assessment and standardized imaging are needed.

### Declaration of generative AI and AI-assisted technologies in the writing process

AI-assisted tools were used only for language editing. All content was reviewed and approved by the authors, who take full responsibility for the content of the manuscript.

## Conflicts of interest

None disclosed.
